# Ultrasound Shear-Wave and Strain Elastography for Differentiating Morton’s Neuroma from Intermetatarsal Bursitis

**DOI:** 10.3390/life16101628

**Published:** 2026-09-29

**Authors:** Giovanni Sussan, Antonio Corso, Raimondo Angelone, Giacomo Negro, Giovanni Sciarretta, Alberto Crimì, Carlo Biz, Pietro Ruggieri, Emilio Quaia, Filippo Crimì

**Affiliations:** 1 Institute of Radiology, Department of Medicine-DIMED, University of Padova, Via Giustiniani 2, 35128 Padova, Italy; 2 Orthopedics and Orthopedic Oncology Unit, Department of Surgery, Oncology and Gastroenterology DiSCOG, University of Padova, Via Giustiniani 2, 35128 Padova, Italy

**Keywords:** Morton’s neuroma, intermetatarsal bursitis, shear-wave elastography, strain elastography, ultrasound

## Abstract

**Background**: Ultrasound (US) is a well-established imaging modality for the diagnosis of Morton’s neuroma (MN), although differentiating MN from intermetatarsal bursitis (IMB) may be challenging in some cases. US elastography, including strain elastography (SE) and shear wave elastography (SWE), enables the assessment of tissue stiffness. Because MN is predominantly composed of fibrotic tissue whereas IMB contains fluid, we hypothesized that elastography could improve the differentiation between these two conditions. **Methods**: Fifty consecutive patients (43 women) with clinically suspected MN involving 71 feet were prospectively enrolled. All patients underwent both US and MRI, independently interpreted by two radiologists. Patients with imaging findings suspicious for MN on either modality underwent surgery, with histopathology serving as the reference standard. Cases not meeting surgical criteria were classified as IMB based on imaging and clinical follow-up. During US examination, SE and SWE were performed. The Strain Ratio (SR), SWE lesion stiffness, subcutaneous tissue stiffness, and the SWE lesion-to-subcutaneous tissue stiffness ratio (hereafter, SWE ratio) were recorded. Elastographic parameters were compared between MN and IMB, and their diagnostic performance was evaluated using receiver operating characteristic (ROC) curve analysis. **Results**: MN demonstrated significantly higher SR and SWE stiffness than IMB. The SWE ratio achieved the highest diagnostic performance, with an AUC significantly greater than those of SWE lesion stiffness (0.915 vs. 0.718, *p* = 0.004) and SR (0.915 vs. 0.719, *p* = 0.002). Using a cut-off value >1.5, the SWE ratio yielded 79.2% sensitivity, 88.9% specificity, and 81.7% overall accuracy. **Conclusions**: US elastography, particularly the SWE ratio, may represent a valuable adjunct to conventional US for differentiating MN from IMB, improving the diagnostic performance of US.

## 1. Introduction

Morton’s neuroma (MN) is an entrapment neuropathy affecting the common plantar digital nerve within the intermetatarsal spaces of the forefoot. MN is particularly common in middle-aged women, often associated with high-heeled or narrow toe-box footwear, and most frequently affects the third intermetatarsal space, followed by the second; more than one lesion in the same foot, and bilateral involvement, can also occur [1].

The development of MN is thought to result from chronic repetitive mechanical stress within the intermetatarsal space [2].

Clinically, patients typically present with burning or sharp pain localized to the affected intermetatarsal space, often radiating to the adjacent toes and associated with paresthesia, tingling, or numbness [3].

Symptoms are usually exacerbated by weight-bearing activities or tight footwear and are relieved by rest and shoe removal [4]. Among the available clinical examination maneuvers, the thumb–index finger squeeze test has been reported to be one of the most sensitive screening tests for MN [5,6].

Mulder’s maneuver, performed by mediolateral compression of the metatarsal heads combined with plantar pressure over the affected intermetatarsal space, remains diagnostically valuable when it reproduces the patient’s typical pain together with a palpable click [2].

Musculoskeletal US is widely considered the first-line imaging modality for the evaluation of suspected MN [7]. The examination is typically performed using a high-frequency linear transducer through either a dorsal or plantar approach, with the patient in a relaxed supine or seated position [8]. Sonographically, MN usually appears as a well-defined hypoechoic, non-compressible focal thickening of the common plantar digital nerve, most frequently located near the point of nerve bifurcation [8]. One of the main differential diagnoses of MN is intermetatarsal bursitis (IMB), which frequently presents with similar clinical symptoms [9]. IMB is characterized by inflammation of the intermetatarsal bursa located between the metatarsal heads, dorsal to the deep transverse metatarsal ligament, resulting in forefoot pain that may clinically mimic MN [9]. Although conventional US is highly accurate for diagnosing MN, distinguishing MN from IMB may occasionally be challenging, even with modern high-resolution US systems [8,10]. Dynamic evaluation is particularly useful in this setting: whereas MN typically appears as a solid, hypoechoic, non-compressible lesion, IMB is generally visualized as a compressible hypoechoic fluid-containing structure within the intermetatarsal space [11].

US elastography is an advanced imaging technique that complements conventional B-mode US by providing information on tissue mechanical properties, particularly tissue stiffness [12,13,14].

Because MN is characterized by extensive intraneural and perineural fibrosis, it is expected to demonstrate greater tissue stiffness than IMB, which is predominantly fluid-filled [2,9]. Therefore, US elastography may improve the differentiation between these two entities when conventional B-mode findings are equivocal [12,13].

We hypothesized that US elastography [12,13], by quantifying the greater tissue stiffness expected in the fibrotic MN compared with the fluid-filled IMB [2,9], could improve the differentiation between these two entities beyond conventional B-mode US, a clinically meaningful distinction with direct treatment implications [10], particularly in equivocal cases where MRI, despite its complementary diagnostic value [4], remains impractical as a routine first-line tool. The aim of the present study was to evaluate the diagnostic performance of SE and SWE for the identification and characterization of MN compared to IMB.

## 2. Materials and Methods

### 2.1. Patients

This prospective, comparative, single-center observational cohort study was approved by the local Institutional Ethics Committee (see Institutional Review Board Statement), and represents a sub-study of a study comparing US and MRI for MN detection [15].

Written informed consent was obtained from all participants before enrollment, in accordance with the principles of the Declaration of Helsinki.

Patients with a clinical suspicion of MN were consecutively and prospectively recruited over a 12-month period, from July 2024 to June 2025. Eligible participants were adults (≥18 years) presenting with persistent pain localized to the second or third intermetatarsal space for at least 6 months, without involvement of the first or fourth intermetatarsal spaces, and reporting symptom exacerbation while wearing shoes. Exclusion criteria included recent foot trauma, clinical signs of active infection, previous forefoot surgery, rheumatologic disease, diabetes mellitus, or peripheral neuropathy.

All patients underwent a standardized clinical evaluation performed by a senior foot and ankle orthopedic surgeon. Eligibility required a positive Mulder test in at least one of the two suspected intermetatarsal spaces. Following clinical assessment, all patients underwent US, including SE and SWE, and MRI of the forefoot on the same day at the Department of Radiology of our institution.

The imaging examinations were independently interpreted by two experienced musculoskeletal radiologists, each evaluating only one imaging modality. Both radiologists were aware of the clinical suspicion of MN and the affected intermetatarsal space but were blinded to the findings of the other imaging modality. In patients with bilateral clinical suspicion, both feet were examined, and each foot was considered an independent statistical unit. Based on the imaging findings, each examined foot was classified as either MN or IMB.

MRI was performed in addition to US to provide an independent, blinded confirmation of this imaging-based classification, rather than to serve as a diagnostic comparator to US elastography, which was outside the scope of this study.

Following imaging evaluation, all patients were referred to the same senior foot and ankle orthopedic surgeon for treatment. Feet with imaging findings consistent with MN underwent surgical excision, whereas those classified as IMB received conservative treatment consisting of nonsteroidal anti-inflammatory drugs (NSAIDs) and metatarsal pads.

The final diagnosis was established using different reference standards according to the treatment received. For surgically treated feet, the diagnosis of MN was confirmed by histopathological examination of the excised specimen obtained within 15 days after imaging. For conservatively treated feet, the diagnosis of IMB was confirmed clinically by complete resolution of symptoms at the 6-month follow-up. Based on these reference standards, each evaluated foot was assigned to either the MN or the IMB group for the final statistical analysis.

### 2.2. Imaging Techniques

MRI of the forefoot was performed using a 1.5-T scanner (Magnetom Avanto, Siemens, Erlangen, Germany) equipped with dedicated foot coils, with patients positioned supine. The imaging protocol, based on the recommendations of the European Society of Skeletal Radiology (ESSR), comprised T1-weighted and fat-saturated T2-weighted sequences acquired in the axial, coronal, and sagittal planes; no intravenous contrast medium was administered.

On MRI, Morton’s neuroma was defined by the presence of a well-circumscribed lesion in the plantar aspect of the intermetatarsal space near the metatarsal heads, showing hypointense signal intensity on both T1- and T2-weighted sequences, whereas the presence of fluid signal intensity within the intermetatarsal space was considered indicative of IMB [1].

As with US, MRI images were interpreted independently by a musculoskeletal radiologist assigned exclusively to this imaging modality and blinded to the findings of the US examination.

**Figure 1:**
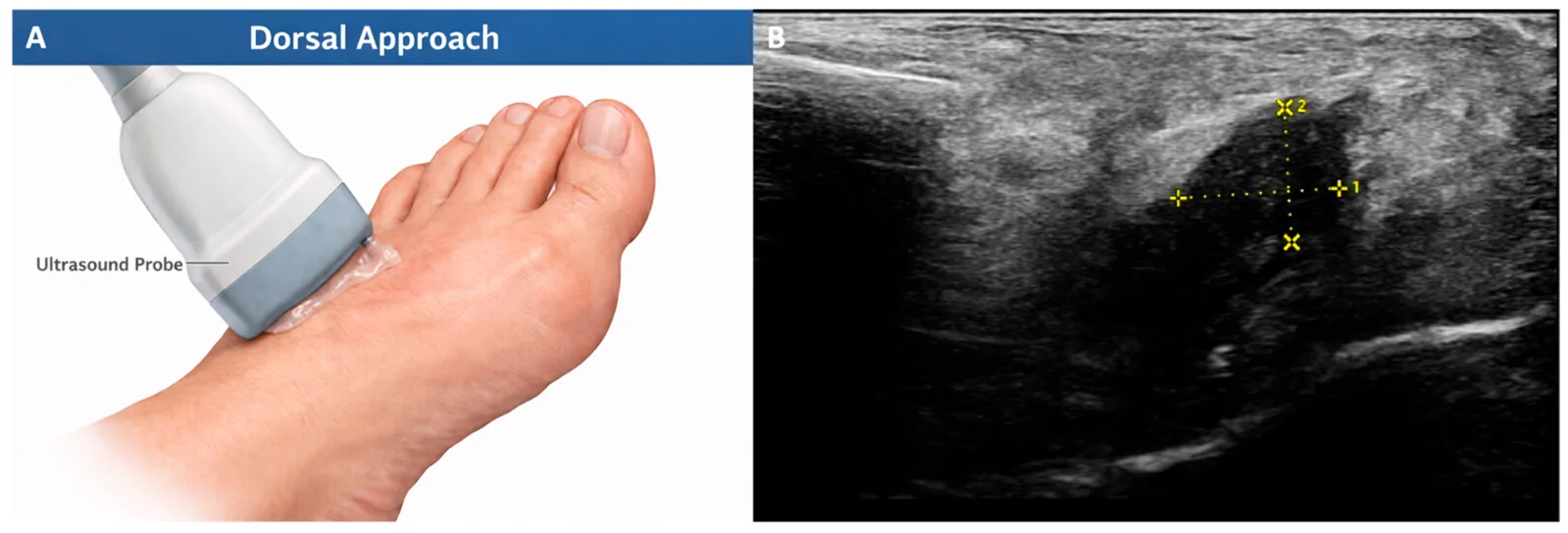
(**A**) Schematic drawing illustrating the dorsal US approach, with the probe positioned along the intermetatarsal space. (**B**) Corresponding B-mode US image of an intermetatarsal space obtained from a dorsal approach, demonstrating a Morton’s neuroma (calipers).

US examinations were performed using a high-resolution US system available at the Department of Radiology of our institution (RS85 Prestige, Samsung Medison Co., Ltd., Seoul, Republic of Korea), equipped with a 2–14 MHz linear-array transducer. The US assessment was conducted using both a dorsal approach, primarily focused on the intermetatarsal space clinically suspected of harboring Morton’s neuroma (Figure 1), and a plantar approach combined with lateral compression of the forefoot to allow dynamic evaluation of Mulder’s sign.

During the US examination, the location, morphology, margins, echotexture, maximum diameter, compressibility, Power Doppler signal, continuity with the plantar digital nerve, and the presence of Mulder’s sign were assessed to establish a final diagnosis of MN or IMB, as previously described [1].

Moreover, US elastography, including both SE and SWE, was performed in all cases to provide complementary information on tissue stiffness and biomechanical properties. For SE, the lesion was first identified on the B-mode grayscale image. The US probe was then placed dorsally over the selected intermetatarsal space and gently compressed at a frequency of two to three compression–decompression cycles per second, while being kept perpendicular to the skin with light pressure. A quality indicator displayed on the screen was maintained in the green range to ensure adequate elastographic image quality. Once the color elastography map had been obtained, a ROI encompassing the lesion was selected for stiffness assessment. ROI placement was based on qualitative visual assessment of the B-mode image and the corresponding SE color map, with the ROI positioned within the most homogeneous, solid-appearing portion of the lesion, avoiding cystic areas or regions affected by visible artifacts.

**Figure 2:**
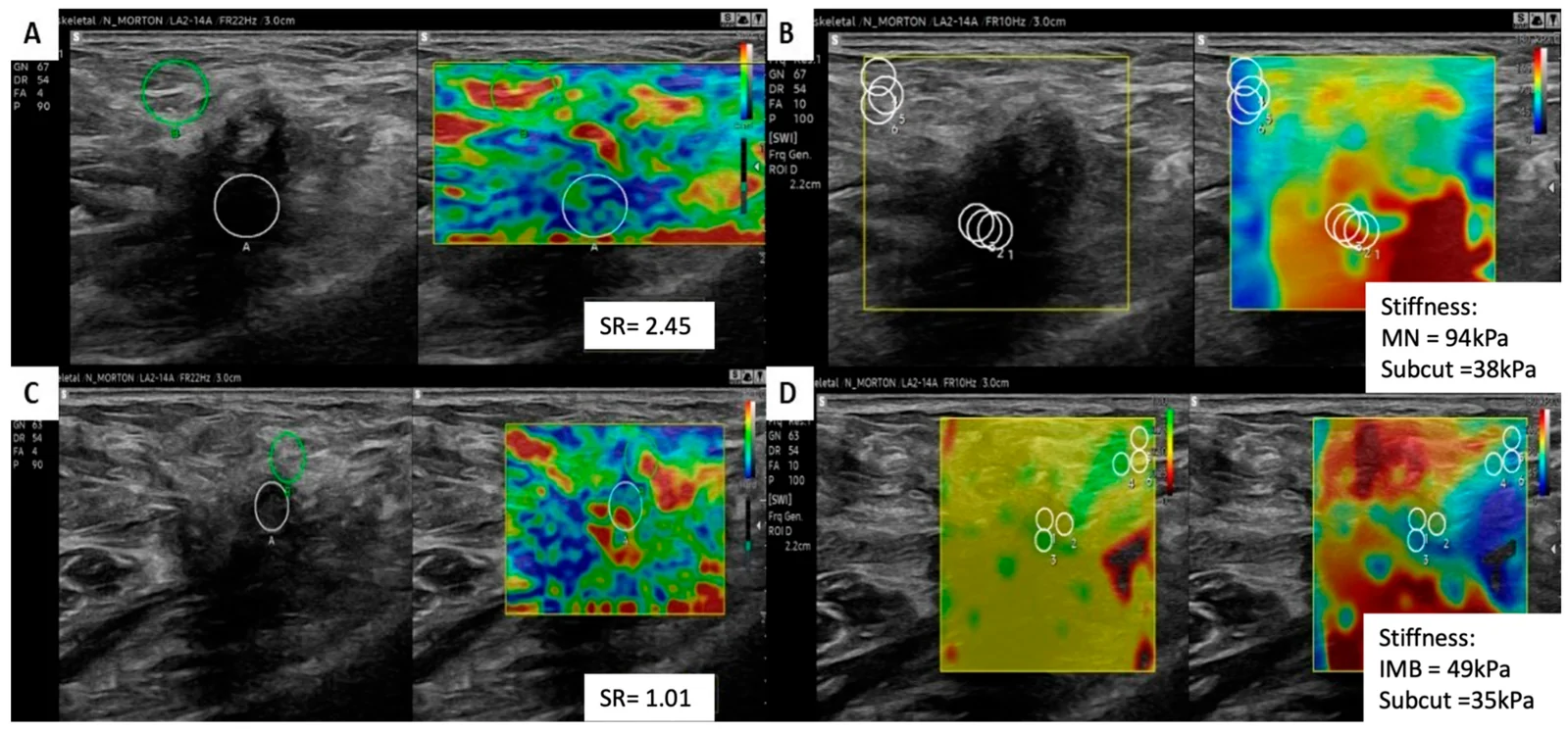
Strain and shear-wave ultrasound images of Morton’s neuroma (MN; (**A**,**B**)) and intermetatarsal bursitis (IMB; (**C**,**D**)). The strain ratio (SR) was 2.45 for MN (**A**) and 1.01 for IMB (**C**). Shear-wave ultrasound showed greater lesion stiffness in MN (94 kPa; (**B**)) than in IMB (49 kPa; (**D**)), whereas subcutaneous tissue stiffness was comparable (38 vs. 35 kPa, respectively).

On SE, stiff tissues are displayed in blue, soft tissues in red, and tissues with intermediate stiffness in green to blue-green. As a reference, another ROI was placed within the subcutaneous fat tissue, avoiding abnormalities of the plantar fat pad, and the Strain Ratio (SR) was calculated (Figure 2).

For SWE, similarly to SE, the US probe was positioned dorsally over the intermetatarsal space, and the lesion was identified using B-mode. Once a clear view of the intermetatarsal space had been obtained, the SWE mode was activated, applying minimal pressure to minimize tissue precompression. The US probe was maintained in the same position for at least 10 s to allow stabilization of the elastography color map. Three circular ROIs with 0.2 cm diameter were placed within the lesion on the elastography map following the same qualitative criteria described for SE to obtain three elasticity measurements, automatically calculated by the US system and expressed in kPa, from which the mean lesion stiffness was calculated (Figure 2). Similarly, three ROIs were placed within the subcutaneous tissue, avoiding areas affected by compression artifacts, which could result in increased stiffness, to obtain the mean stiffness of the reference tissue. Finally, a stiffness ratio was calculated by dividing the mean stiffness of the lesion by that of the subcutaneous tissue.

### 2.3. Surgical Operations and Histopathology

Surgical excision of Morton’s neuroma was performed in all indicated cases by the same senior foot and ankle orthopedic surgeon, who had extensive experience in minimally invasive foot and ankle surgery. A minimally invasive dorsal approach was used in all procedures.

Patients were positioned supine, and an ankle block was administered before application of an ankle tourniquet. A 2–2.5 cm longitudinal dorsal incision was made over the affected intermetatarsal space (third or fourth). The subcutaneous tissues were bluntly dissected, and, after achieving adequate hemostasis, dissection was continued into the deep intermetatarsal space while carefully preserving the dorsal digital nerves. The common digital nerve and the neuroma were identified, and the distal digital nerve branches were transected. The common digital nerve, including the neuroma, was then grasped with a Kocher clamp. After that the next was mobilized as proximally as possible and excised.

Following tourniquet release, meticulous hemostasis was achieved, and the surgical field was irrigated with sterile saline solution. Skin closure was performed using absorbable 3-0 sutures, followed by application of a sterile dressing. The excised specimens were fixed in phosphate-buffered formalin, embedded in paraffin, sectioned, and processed for histopathological analysis. Histological evaluation was performed using hematoxylin–eosin and trichrome staining to assess the characteristic features of Morton’s neuroma.

Postoperatively, patients were allowed to ambulate as tolerated from the day of discharge while wearing a heel weight-bearing orthopedic shoe. Sutures were removed at the routine 2-week follow-up visit.

### 2.4. Statistical Analysis

The collected data are presented, as appropriate, as percentages, medians with interquartile ranges (IQR), or means ± standard deviation (SD). The Kolmogorov–Smirnov test was used to assess the normality of continuous variables. For the statistical analyses, the examined feet were classified into two groups according to the final diagnosis: MN or IMB.

Differences in SR, lesion stiffness, and the SWE ratio were evaluated using Student’s *t*-test for normally distributed variables and the Wilcoxon–Mann–Whitney U test for non-normally distributed variables.

Receiver operating characteristic (ROC) curve analysis was performed for SR, lesion stiffness, and the SWE ratio using the final diagnosis (MN or IMB) as the reference standard. The optimal cut-off value was determined using the Youden index. The areas under the ROC curves (AUCs) were calculated and compared using the DeLong test. For each parameter, sensitivity, specificity, accuracy, positive predictive value (PPV), and negative predictive value (NPV) were calculated.

Statistical significance was defined as a two-sided *p* value < 0.05. All statistical analyses were performed using jamovi (version 2.7; The jamovi Project, 2026) and MedCalc (version 23.2.0; MedCalc Software Ltd., Ostend, Belgium).

## 3. Results

The analysis included 50 patients (median age, 58 years; IQR, 54–66), of whom 43 were female, representing a total of 71 feet. The final diagnosis was MN in 53 (75%) feet and IMB in 18 (25%) feet.

On SWE, median MN stiffness was significantly higher than that of subcutaneous tissue (89 kPa vs. 48 kPa; *p* < 0.001). Compared with the IMB group, the MN group had significantly higher SR (1.42 vs. 0.90; *p* = 0.0057), lesion stiffness (90.0 kPa vs. 66.5 kPa; *p* = 0.006), and SWE ratio (2.20 vs. 1.07; *p* < 0.001).

**Figure 3:**
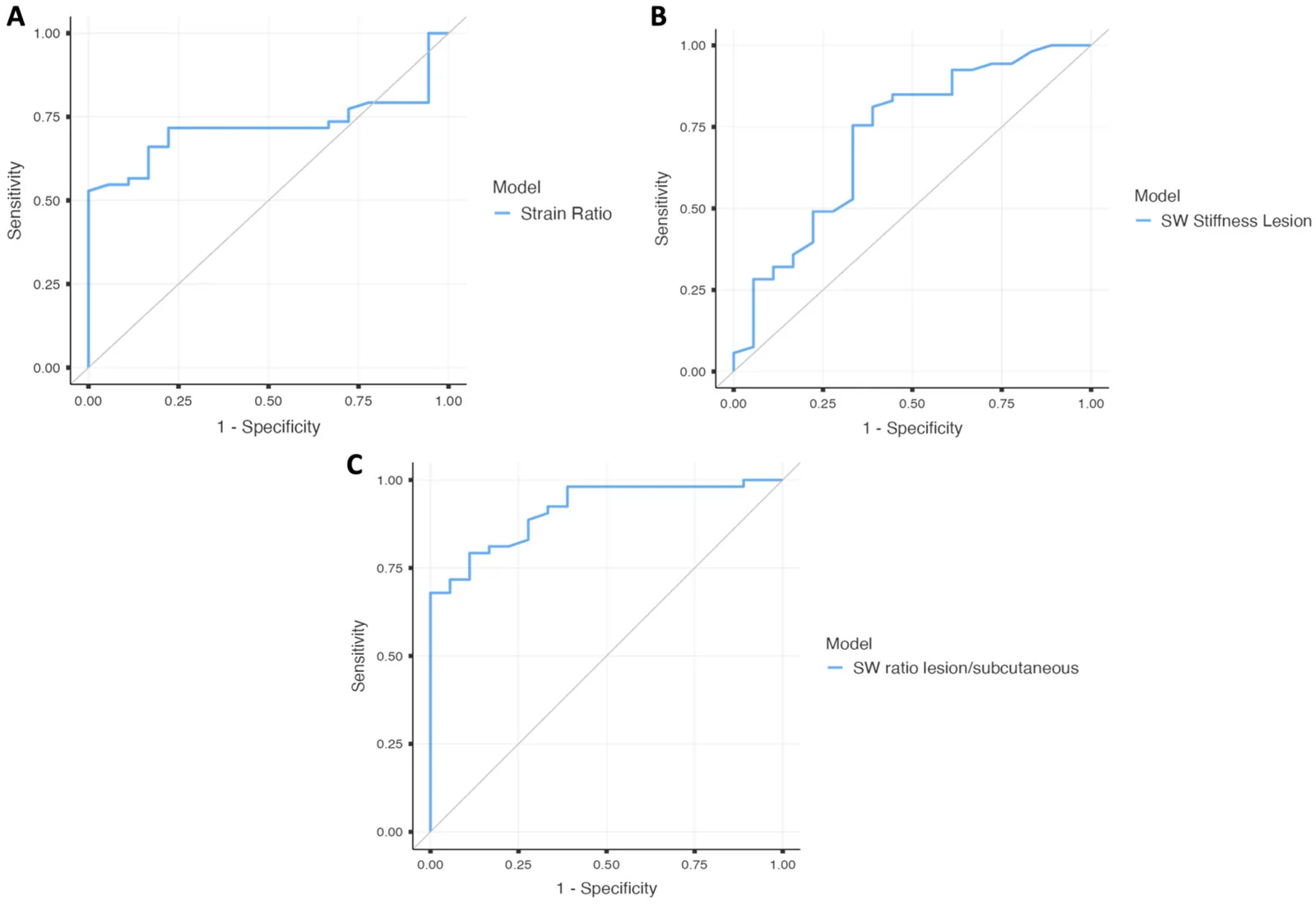
Receiver operating characteristic (ROC) curves for the identification of MN using strain ratio values (**A**), shear wave (SWE) stiffness of the lesion (**B**), and the SW ratio between the lesion and subcutaneous tissue (**C**).

ROC curve analysis showed that SR yielded an AUC of 0.719 (95% CI, 0.603–0.835). Using the Youden index, an optimal cut-off value > 1.375 provided a sensitivity of 53% and a specificity of 100% for the identification of MN (Figure 3A; Table 1).

For SWE lesion stiffness, the AUC was 0.718 (95% CI, 0.569–0.867). The optimal cut-off identified by the Youden index was >73.5 kPa, corresponding to a sensitivity of 81% and a specificity of 61% (Figure 3B; Table 1).

The SWE ratio yielded an AUC of 0.915 (95% CI, 0.849–0.981). At the optimal Youden index cut-off (>1.5), the sensitivity and specificity for identifying MN were 79% and 89%, respectively (Figure 3C; Table 1).

The SWE ratio demonstrated a significantly higher AUC than both SWE lesion stiffness (0.915 [95% CI, 0.849–0.981] vs. 0.718 [95% CI, 0.569–0.867]; *p* = 0.004) and SR (0.915 [95% CI, 0.849–0.981] vs. 0.719 [95% CI, 0.603–0.835]; *p* = 0.002). No statistically significant difference was observed between the AUCs of SWE lesion stiffness and SR (*p* = 0.992).

## 4. Discussion

The present study evaluated the diagnostic performance of SE and SWE for differentiating MN from IMB. To the best of our knowledge, this is the first study to evaluate quantitative SWE for this clinical indication. The main finding was that all elastographic parameters were able to discriminate between the two conditions. However, although SR and absolute lesion stiffness measured by SWE showed comparable diagnostic performance, normalization of lesion stiffness to the adjacent subcutaneous tissue significantly improved diagnostic accuracy, emerging as the best-performing elastographic parameter. Interestingly, SE-derived SR and absolute SWE lesion stiffness demonstrated almost identical discriminative ability. Despite being based on different physical principles, both techniques yielded comparable ROC curve performance, suggesting that they capture similar biomechanical information when lesion stiffness is evaluated as an isolated parameter. From a practical perspective, these findings indicate that neither semiquantitative strain measurements nor absolute SWE stiffness values provide a clear diagnostic advantage over the other.

In contrast, the SWE ratio demonstrated significantly greater diagnostic performance than both SR and absolute lesion stiffness. This finding suggests that the use of an internal reference tissue reduces the variability associated with absolute elasticity measurements. Individual differences in foot soft-tissue composition, tissue elasticity, probe pressure, and acquisition conditions may influence absolute stiffness values, whereas normalization to adjacent subcutaneous tissue provides a more stable estimate of the intrinsic mechanical properties of the lesion. This approach is analogous to the use of tissue ratios in elastography of other anatomical regions, where internal normalization has been shown to improve measurement robustness by minimizing inter-individual and technical variability. The balanced sensitivity and specificity achieved by the SWE ratio further support its potential clinical usefulness for differentiating MN from IMB. In particular, the cut-off value of 1.5 identified by the Youden index showed the highest overall diagnostic performance and may represent a practical threshold for routine clinical use, providing supportive information in cases where conventional B-mode findings are indeterminate, to be interpreted alongside clinical and conventional imaging findings rather than in isolation, given its imperfect sensitivity and specificity.

It should be emphasized that, for both SR and SWE, despite the statistically significant differences in values between MN and IMB, a slight overlap between the groups was observed. This overlap was reduced when the SWE ratio was used. It was probably caused by interpatient variability in the measurements, which was partially corrected by the intrapatient normalization provided by the SWE ratio.

To the best of our knowledge, only one previous study has investigated US elastography in MN. Ormeci et al. evaluated SE and reported significantly higher elasticity and SR values in MN than in the adjacent normal tissue of the same intermetatarsal space, findings that are consistent with our results obtained using SWE [16].

However, unlike the present study, they did not compare MN with IMB, which represents one of the most common and clinically relevant differential diagnoses encountered during forefoot US [16].

Consequently, our study extends the available evidence by demonstrating that quantitative SWE, particularly when normalized to the adjacent subcutaneous tissue, provides excellent discriminatory performance between these two entities.

US is a well-established imaging modality for the diagnosis of MN [1,17].

A meta-analysis published in 2015 demonstrated that US and MRI have comparable pooled sensitivity for detecting MN (91% vs. 90%), although MRI showed higher pooled specificity (100% vs. 85%) [18].

Nevertheless, recent evidence has highlighted that differentiating MN from IMB based solely on conventional imaging findings remains challenging, particularly when the two conditions coexist or exhibit overlapping morphological features [10].

Therefore, additional quantitative imaging biomarkers capable of improving diagnostic confidence are desirable.

Given the excellent diagnostic performance of conventional ultrasound and the overlap in elastographic measurements between MN and IMB, SE and SWE should currently be regarded as complementary tools, with prospective studies needed to establish their incremental value and validate the proposed cut-offs.

### Limitations

This was a single-center study, which may limit the external validity of our findings; future multicenter studies with larger cohorts are warranted to confirm and generalize our results.

An important limitation is the use of different reference standards—histopathology for MN and clinical follow-up for IMB—which introduces potential verification bias and limits between-group comparisons. This approach reflects ethical constraints, as surgery solely for histopathological confirmation was not permitted in patients with IMB in the absence of expected clinical benefit. Although full US–MRI concordance and subsequent symptom resolution supported the IMB diagnosis, they cannot replace histopathological confirmation. Nevertheless, the use of clinical follow-up as the reference standard for IMB is consistent with previous studies.

All US and elastographic examinations were performed by a single experienced operator; therefore, formal inter-observer reproducibility of SR and SWE measurements could not be assessed. Although methodological precautions were adopted to minimize pressure-related artifacts—including a standardized acquisition protocol, averaging of multiple ROI measurements, and normalization of lesion stiffness to the adjacent subcutaneous tissue—future studies involving multiple independent operators are warranted to confirm the reproducibility of these measurements, given the known susceptibility of superficial soft-tissue stiffness values to transducer precompression.

Absolute stiffness values (kPa) and the resulting cut-offs may vary across US systems from different vendors due to proprietary beam-forming and post-processing algorithms. Although the SWE ratio is expected to be comparatively more robust to this source of variability, since both measurements are obtained with the same system within the same acquisition, external validation of the proposed cut-off values on different US platforms is warranted before their broader clinical adoption.

The decision to refer patients to surgery, and thus their assignment to the histologically confirmed MN group, was based on conventional B-mode US and MRI findings and clinical presentation, rather than on the elastographic parameters evaluated in this study, which currently have no established role in this clinical decision. While this avoids incorporation bias in the strict sense, it may still enrich the surgical subgroup for cases with more classical presentations, potentially limiting the generalizability of our diagnostic accuracy estimates to genuinely equivocal cases where elastography could be of greatest clinical value.

Finally, the imbalance between the MN (*n* = 53) and IMB (*n* = 18) groups limits the precision of specificity estimates, reflected in the wide 95% confidence intervals in Table 1. Larger, more balanced cohorts are needed in future studies.

## 5. Conclusions

The results of our exploratory study suggest that ultrasound elastography revealed differences in stiffness between MN and IMB, indicating that it may be a valuable adjunct to conventional B-mode ultrasound for differentiating these two entities. Among the elastographic parameters assessed, the SWE ratio showed the highest diagnostic performance, out-performing both SR and absolute SWE lesion stiffness, and may serve as a practical and reproducible tool to improve diagnostic confidence in equivocal cases, complementing rather than replacing conventional B-mode assessment. These findings should be interpreted in light of the limitations discussed above. Further studies, including external validation across different ultrasound platforms and larger, multi-center cohorts, are warranted to confirm these findings and support their integration into routine clinical practice.

## Figures and Tables

**Table 1:** Diagnostic performance for the identification of Morton’s neuroma using strain ratio, shear wave (SW) stiffness of the lesion, and the SW ratio between the lesion and subcutaneous tissue, with cut-off values determined by the Youden index from the ROC curves.

	Strain Ratio	SWE Stiffness Lesion	SWE Ratio Lesion/Subcutaneous
**Sensitivity**	53% (95% CI: 39–67%)	81% (95% CI: 68–91%)	79% (95% CI: 66–89%)
**Specificity**	100% (95% CI: 81–100%)	61% (95% CI: 36–83%)	89% (95% CI: 65–99%)
**Positive Predictive Value**	100% (95% CI: 88–100%)	86.00% (95% CI: 77–92%)	95.45% (95% CI: 85–99%)
**Negative Predictive Value**	42% (95% CI: 35–9%)	52% (95% CI: 36–68%)	59% (95% CI: 46–72%)
**Accuracy**	65% (95% CI: 53–76%)	76% (95% CI: 64–85%)	82% (95% CI: 71% –90%)

CI = Confidence Interval.

## Data Availability

The data that support the findings of this study are available from the corresponding author upon reasonable request.

## References

[1] Mak M.S., Chowdhury R., Johnson R. (2021). Morton’s Neuroma: Review of Anatomy, Pathomechanism, and Imaging. Clin. Radiol..

[2] Wu K.K. (1996). Morton’s Interdigital Neuroma: A Clinical Review of Its Etiology, Treatment, and Results. J. Foot Ankle Surg..

[3] Campbell C.M., Diamond E., Schmidt W.K., Kelly M., Allen R., Houghton W., Brady K.L., Campbell J.N. (2016). A Randomized, Double-Blind, Placebo-Controlled Trial of Injected Capsaicin for Pain in Morton’s Neuroma. Pain.

[4] Fazal M.A., Khan I., Thomas C. (2012). Ultrasonography and Magnetic Resonance Imaging in the Diagnosis of Morton’s Neuroma. J. Am. Podiatr. Med. Assoc..

[5] Dando C., Cherry L., Jones L., Bowen C. (2017). The Clinical Diagnosis of Symptomatic Forefoot Neuroma in the General Population: A Delphi Consensus Study. J. Foot Ankle Res..

[6] Mahadevan D., Venkatesan M., Bhatt R., Bhatia M. (2015). Diagnostic Accuracy of Clinical Tests for Morton’s Neuroma Compared With Ultrasonography. J. Foot Ankle Surg..

[7] Xu Z., Duan X., Yu X., Wang H., Dong X., Xiang Z. (2015). The Accuracy of Ultrasonography and Magnetic Resonance Imaging for the Diagnosis of Morton’s Neuroma: A Systematic Review. Clin. Radiol..

[8] Fenech M. (2024). Sonographic Anatomy and Technique to Image the Plantar Digital Nerves and Aid Identification of a Morton’s Neuroma. Ultrasound.

[9] Larsen S.B., Søgaard S.B., Nielsen M.B., Torp-Pedersen S.T. (2023). Diagnostic Considerations of Intermetatarsal Bursitis: A Systematic Review. Diagnostics.

[10] Larsen S.B., Offersen C.M., Dyrberg E., Johansen J.K., Lange N.B., Bech B.H., Nielsen M.B., Torp-Pedersen S.T. (2025). Morton’s Neuroma or Intermetatarsal Bursitis—A Prospective Diagnostic Study of Intermetatarsal Pain. Diagnostics.

[11] Ganguly A., Warner J., Aniq H. (2018). Central Metatarsalgia and Walking on Pebbles: Beyond Morton Neuroma. Am. J. Roentgenol..

[12] Schneebeli A., Fiorina I., Bortolotto C., Barbero M., Falla D., Cescon C., Raciti M.V., Tarantino F., Preda L. (2021). Shear Wave and Strain Sonoelastography for the Evaluation of the Achilles Tendon during Isometric Contractions. Insights Imaging.

[13] Drakonaki E.E., Allen G.M., Wilson D.J. (2012). Ultrasound Elastography for Musculoskeletal Applications. Br. J. Radiol..

[14] Bercoff J., Tanter M., Fink M. (2004). Supersonic Shear Imaging: A New Technique for Soft Tissue Elasticity Mapping. IEEE Trans. Ultrason. Ferroelectr. Freq. Control.

[15] Biz C., Crimì F., Sciaretta G., Crimì A., Dimaggio A., Corso A., Angelone R., Quaia E., Ruggieri P. (2026). Dynamic ultrasound (DUS) versus magnetic resonance imaging (MRI) for the diagnosis of Morton’s neuroma: A prospective comparative study. Foot Ankle Surg..

[16] Ormeci T., Güler O., Malkoc M., Keskinbora M., Güngören F.Z., Mahirogulları M. (2016). Diagnostic Value of Elastography in the Diagnosis of Intermetatarsal Neuroma. J. Foot Ankle Surg..

[17] Santiago F.R., Muñoz P.T., Pryest P., Martínez A.M., Olleta N.P. (2018). Role of Imaging Methods in Diagnosis and Treatment of Morton’s Neuroma. World J. Radiol..

[18] Bignotti B., Signori A., Sormani M.P., Molfetta L., Martinoli C., Tagliafico A. (2015). Ultrasound versus Magnetic Resonance Imaging for Morton Neuroma: Systematic Review and Meta-Analysis. Eur. Radiol..

